# How many doses and what type of antibiotic should be used as systemic antibiotic prophylaxis in primary hip and knee arthroplasty? A register-based study on 301,204 primary total and hemi- hip and total knee arthroplasties in Norway 2005–2023

**DOI:** 10.2340/17453674.2025.43003

**Published:** 2025-03-04

**Authors:** Olav LUTRO, Marianne Bollestad TJØRHOM, Tesfaye Hordofa LETA, Jan-Erik GJERTSEN, Geir HALLAN, Trond BRUUN, Marianne WESTBERG, Tina Strømdal WIK, Christian Thomas POLLMANN, Stein Håkon LYGRE, Ove FURNES, Lars ENGESÆTER, Håvard DALE

**Affiliations:** 1 Department of Medicine, Stavanger University Hospital, Stavanger; 2 The Norwegian Arthroplasty Register, Department of Orthopaedic Surgery, Haukeland University Hospital, Bergen; 3 Faculty of Health Studies, VID Specialized University, Bergen; 4 The Norwegian Hip Fracture Register, Department of Orthopaedic Surgery, Haukeland University Hospital, Bergen; 5 Department of Clinical Medicine, University of Bergen, Bergen; 6 Department of Clinical Science, University of Bergen, Bergen; 7 Department of Medicine, Haukeland University Hospital, Bergen; 8 Division of Orthopedic Surgery, Oslo University Hospital, Oslo; 9 Department of Orthopedic Surgery, St. Olavs Hospital, Trondheim; 10 Department of Orthopaedic Surgery, Akershus University Hospital, Lørenskog; 11 Department of Occupational Health, Haukeland University Hospital, Bergen, Norway

## Abstract

**Background and purpose:**

Guidelines for systemic antibiotic prophylaxis (SAP) in arthroplasty surgery vary worldwide from repeated doses to only 1 preoperatively. We aimed to investigate, primarily whether 4 doses reduced the risk of PJI compared with 1 to 3 doses, and secondarily if there was a difference between types of antibiotics.

**Methods:**

Patients reported to the Norwegian Arthroplasty Register and the Norwegian Hip Fracture Register with primary total knee (TKA), total (THA) or hemi- (HA) hip arthroplasty between 2005 and 2023 were included. Cases with 1 to 4 doses of cefalotin (half-life = 45 minutes), cefazolin (90 minutes), cefuroxime (70 minutes), cloxacillin (30 minutes), or clindamycin (180 minutes) were assessed. Primary outcome was 1-year risk of reoperation (adjusted hazard rate ratio; aHRR) for PJI and was estimated by Cox regression analyses. Secondary outcomes were reoperation for PJI and reoperation for any cause with follow-up of up to 19 years. Non-inferiority analyses and propensity score matching with subsequent Kaplan–Meier analyses were performed with a predetermined non-inferiority margin of 15% (aHRR = 1.15).

**Results:**

301,204 cases were included. Of these, 3,388 (1.1%) were reoperated on for PJI within 1 year. The 1-year incidence of reoperation for PJI was 98/9,760 (1.0%) for 1 dose of SAP, 109/10,956 (0.9%) for 2 doses, 178/18,948 (0.9 %) for 3 doses, and 3,003/261,540 (1.0%) for 4 doses. The 1-year risk (aHRR, 95% confidence interval [CI]) of reoperation for PJI was 1.0 (CI 0.8–1.2), 0.9 (CI 0.8–1.2), and 0.9 (CI 0.9–1.1) for 1, 2, and 3 doses, respectively, compared with 4 doses. The 1-year incidence of reoperation for PJI was 2,162/183,964 (1.2%) for cefalotin, 993/91,159 (1.1%) for cefazolin, 35/4,435 (0.8%) for cefuroxime, 85/9,022 (0.9%) for cloxacillin, and 113/12,624 (0.9%) for clindamycin. Compared with cefazolin, cloxacillin (1.2, CI 1.0–1.6) and cefalotin (1.4, CI 1.2–1.5) had a higher risk of reoperation for PJI, whereas cefuroxime (1.0, CI 0.7–1.4) and clindamycin (1.1, CI 0.9–1.3) had a similar risk.

**Conclusion:**

4 doses of SAP did not reduce the risk of PJI compared with 1 to 3 doses in primary arthroplasty as measured against PJI. Cefazolin, the 1st-generation cephalosporin with the longest half-life, showed the lowest risk of PJI.

PJI is a serious complication after arthroplasty and has been shown to have increased during recent years [1]. Systemic antibiotic prophylaxis (SAP) is established as one of the most important factors to prevent periprosthetic joint infection (PJI) and it is important to know the optimum dose [2]. Guidelines for SAP in arthroplasty vary worldwide from repeated dosages to only 1 preoperatively [3]. In Norway, the national guideline recommends 4 doses of a 1st-generation cephalosporin (cefazolin or cefalotin), starting 30–60 minutes prior to incision [4]. This recommendation is largely based on results from an earlier study from the Norwegian Arthroplasty Register (NAR) on total hip arthroplasties (THA) showing superiority of 4 doses compared with fewer doses [5]. This contrasts with the World Health Organization (WHO) and the US Centers for Disease Control and Prevention (CDC) recommendation against repeated postoperative doses of SAP [3,6].

A multinational register-based observational study on total knee arthroplasty (TKA) reported that 3–4 doses of SAP were most used in Scandinavia, whereas 1–2 doses were preferred in most other countries [7]. Other studies have reported that 1 dose of SAP may be sufficient to prevent PJI [8,9].

The ecological consequences of widespread use of antibiotics and the influence on skin and gut microbiota has led to a growing awareness concerning antibiotic stewardship. In Norway, antibiotic governance is considered of the highest importance [10]. National guidelines for antibiotic use, including SAP, are regularly revised to find the optimal, evidence-based balance between patient safety and ecological sustainability. In addition, repeated doses of SAP after surgery potentially add challenges in patient safety and logistics, and increase healthcare costs [11].

Cephalosporins are generally well tolerated regarding allergy, and have low liver and kidney toxicity, and few or no adverse effects such as clostridium colitis when used as prophylaxis [12,13]. However, all antibiotics administered could affect the normal bacterial flora of the skin and gut, and fewer dosages would be preferable [14].

Joint replacement surgery has evolved over the years. The length of hospital stay is reduced, and outpatient surgery is emerging, which is why regimens for SAP that are easier to administer are desirable [15].

We aimed to investigate, first, whether 4 doses of SAP reduced risk of PJI compared with 1 to 3 doses and, second, if there was a difference between types of antibiotics.

## Methods

### Study design

This register-based observational cohort study is reported in accordance with the STROBE statement, with additional considerations according to the CONSORT statement with the multiple-arm, parallel-group extension [16-18].

### Setting

The NAR has gathered data on THAs since 1987, and on TKAs since 1994 [19]. The Norwegian Hip Fracture Register (NHFR) was started in 2005 and collects data on hip fracture treatment, including hemi-arthroplasties (HA) [20]. The period of inclusion for the present study was 2005 to 2023. Data on arthroplasties (THA, TKA, and HA) from the 2 registers was merged into 1 dataset. SAP has been reported to NAR and NHFR individually and uniformly since inception. Information on patient characteristics, indication for and type of arthroplasty, implants used, and prophylactic measures are reported immediately after surgery. Thus, intention to treat is reported for postoperative interventions such as repeated doses of SAP. The type of antibiotics, the number of doses, and time interval from surgery to the last dose are reported; however, the exact timing of the first dose SAP is not reported.

All reoperations are reported to the registers in the same manner. The reported cause of reoperation is based on pre- and intraoperative assessment. The PJI diagnosis is not corrected according to intraoperative bacterial samples. Nonetheless, the accuracy of the diagnosis PJI as cause for reoperation has been found to be 87% [21].

The completeness of data in the NAR, validated against the Norwegian Patient Register, is 97% reporting of primary THAs, 91% reporting of any reoperation after THA, 97% reporting of primary TKAs, and 93% reporting of any reoperation after TKA [19]. Completeness in the NHFR is 92% reporting of primary HAs and 88% reporting of any reoperation after HA [19]. The coverage of Norwegian hospitals is 100% for both registers, as is the completeness of reporting of deaths collected from Statistics Norway.

### Study population

In our study, we included patients over the age of 18. SAP was defined as antibiotics given preoperatively and, in the case of subsequent dosing, a maximum of 4 doses within 24 hours of surgery. The SAP regimens analyzed were 1, 2, 3, or 4 doses of the 5 most common antibiotics used (cefalotin, cefazolin, cefuroxime, cloxacillin, or clindamycin). Cefalotin (T_1/2_ 45 minutes) and cloxacillin (T_1/2_ 30 minutes) have the shortest half-life of the antibiotics included in our analyses. Most patients received 4 doses of SAP, and cefalotin and cefazolin were by far the most frequently used antibiotics.

### Outcomes

The primary outcome was reoperation for PJI at 1 year. Secondary outcomes were reoperation for PJI or reoperation for any cause at up to 19 years. Reoperation for PJI was defined as any reoperation (soft tissue debridement with or without revision, with exchange or removal of prosthesis components) with infection reported as the cause.

### Statistics

4 doses of SAP comprised the reference, with which 1, 2, or 3 doses were compared. In addition, we compared the different types of antibiotics with the presently most common cefazolin as the reference.

We performed Kaplan–Meier (KM) and Cox regression analyses. Patients were censored at reoperation for any other cause (in the case of PJI), death, emigration, or end of follow-up (December 31, 2023). Follow up was 0 to 1 (–19) years, with sensitivity analyses of patients with complete 1-year follow-up only.

To visualize the relevant confounders for adjustment, we created a directed acyclic graph (DAG) using DAGitty (https://www.dagitty.net/) [22] (see Appendix). Adjusted hazard rate ratio (aHRR) as an expression of risk was estimated, adjusted for sex, age, ASA class, indication for arthroplasty, and year of primary arthroplasty. Patients with missing information were excluded. The only information that was missing was ASA class, which was assumed to be missing completely at random [23].

In addition, we performed non-inferiority analyses for 1, 2, and 3 doses of SAP compared with 4 doses based on 1-year risk of reoperation for PJI, with estimation of absolute numbers needed to harm (NNH), meaning the number of cases exposed to fewer than 4 doses of SAP before an additional reoperation for PJI occurred [24]. The lower the number, the more cases will be harmed by reoperation for PJI. NNH was calculated for the upper 95% confidence interval of the point estimate of risk of reoperation for PJI (as the limit of statistical significance) for 1, 2, and 3 doses of SAP, and compared with the non-inferiority limit. The predetermined non-inferiority limit of increased risk of reoperation due to PJI was set to 15%, in concordance with a large register randomized controlled trial on antibiotic-loaded bone cement in the NAR [25]. A 15% increased risk of reoperation for PJI for 4 doses of SAP equals an NNH of 667 arthroplasty patients.

We also performed propensity score matching, thereby making the SAP dosage groups as comparable as possible. We used 4 doses as a reference group, and compared these with 1, 2, 3, or 1 and 2 doses combined. The groups were matched 1:4, with a matching tolerance of 0.00001. The groups were matched on sex, age, ASA class, indication for arthroplasty, duration of surgery, type of SAP, type of fixation, type of arthroplasty, and year of primary surgery. KM estimates were calculated for the different dosages, pairwise compared with 4 doses of SAP as well as log rank tests.

In sensitivity analyses, we assessed 1, 2, 3, and 4 doses of SAP and 1-year risk of reoperation for PJI in frail (> 75 years, ASA class 3–4, and arthroplasty due to acute—or complication after—hip fracture) versus robust (< 75 years, ASA class 1–2, and arthroplasty due to osteoarthritis) patients, long (> 120 minutes) versus normal (< 120 minutes) duration of surgery, THA, TKA, and HA separately, uncemented versus cemented with antibiotic-loaded bone cement, and each type of SAP separately. We also performed sensitivity analyses adjusted for type of hospital (rural, central, university, private, and special hospital), and with primary hospital as an instrument variable.

The analyses were performed in accordance with guidelines and recommendations for observational studies [23,26].

All statistical analyses were performed using IBM SPSS 29.0 (IBM Corp, Armonk, NY, USA).

### Ethics, registration, data sharing plan, funding, and disclosures

The Regional Ethics Committee approved the study (REK 2024-759220). The registration of data was performed based on patient consent and according to Norwegian and EU data protection rules. No conflict of interest is declared. Data may be accessible upon application to the NAR. Complete disclosure of interest forms according to ICMJE are available on the article page, doi: 10.2340/17453674.2025.43003

## Results

**Figure 1:**
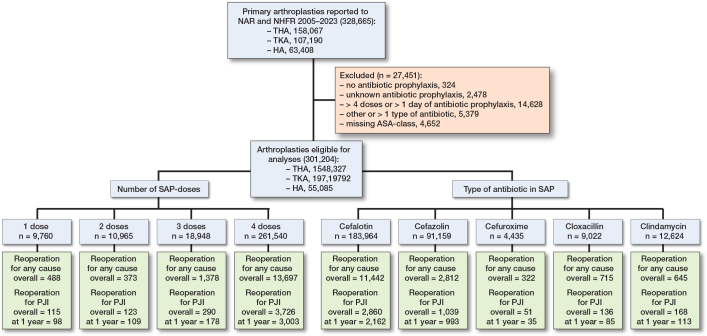
Patient flowchart. Number of arthroplasties included and excluded, and allocation to the different doses and types of systemic antibiotic prophylaxis, with number of primary (1-year reoperation for PJI) and secondary outcomes (overall reoperation for any cause or PJI).

301,204 primary arthroplasties were eligible for analyses (THA 148,327, TKA 97,792, and HA 55,085) (Figure 1). The number of primary arthroplasties included and allocation to the SAP groups with number of primary (1-year reoperation for PJI) and secondary outcomes (overall reoperation for any cause or PJI) are presented in Figure 1.

**Figure 2:**
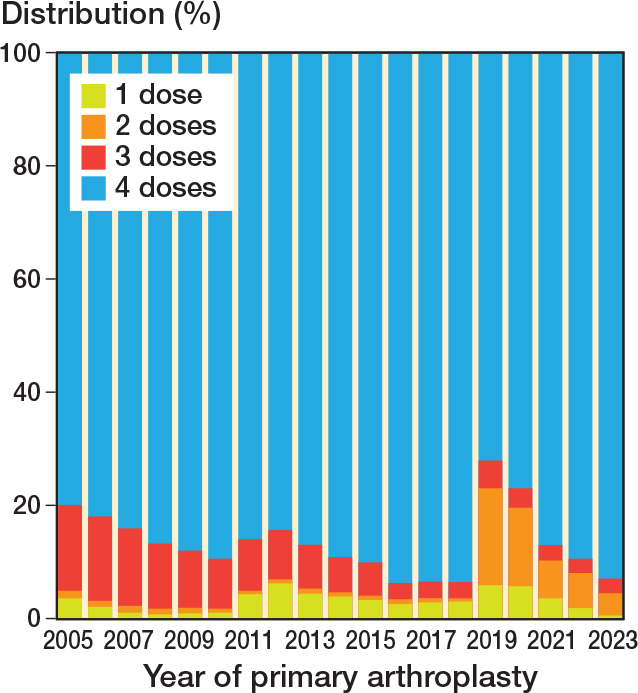
Number of doses of systemic antibiotic prophylaxis by year of primary arthroplasty.

The distribution of patient characteristics to the allocation groups, and number of hospitals that have used the respective dosing and type of SAP regimen during the study period are presented in Table 1. Most patients received 4 doses of SAP, followed by 3, 2, and 1 dose, respectively. Cefalotin was the most used drug, followed by cefazolin. The distribution was similar between the groups except that more women had been given clindamycin. Most hospitals reported several dosing-regimes of SAP. 75/85 of the hospitals used 1 dose, 69/85 used 2 doses, 72/85 used 3 doses, and 84/85 used 4 doses of SAP. 4 doses of SAP were dominant throughout, 3 doses were mostly used early in the study period, whereas 2 doses were increasingly used towards the end of the study period (Figure 2). 1 dose of SAP was used throughout the whole study period in a total of 3% of the cases.

**Table 1:** Patient characteristics with distribution of sex, age, ASA class, indication for arthroplasty, type of arthroplasty, in addition to number of hospitals (N = 85) represented in each allocation group. Values are percentages unless otherwise specified

Risk factor	Doses of SAP				Type of antibiotics in SAP				
	1	2	3	4	Cefalotin	Cefazolin	Cefuroxime	Cloxacillin	Clindamycin
Female	64	63	68	63	64	60	63	62	78
Median age (IQR)	70 (62–77)	72 (64–79)	72 (64–80)	71 (63–79)	71 (63–79)	72 (63–78)	73 (64–80)	71 (63–79)	70 (63–77)
Mean ASA (SD)	2.1 (0.6)	2.2 (0.6)	2.2 (0.7)	2.2 (0.7)	2.1 (0.7)	2.2 (0.6)	2.1 (0.7)	2.1 (0.7)	2.3 (0.6)
Indication for arthroplasty									
Osteoarthritis	78	68	62	67	66	68	62	68	68
Inflammatory disease	2	1	2	2	2	1	3	1	2
Acute or complications after fracture	13	23	31	23	24	23	28	24	22
Other diagnosis	7	7	6	8	8	7	7	7	8
Type of arthroplasty									
THA	59	49	47	49	50	47	50	53	49
TKA	34	31	29	33	31	35	27	30	35
HA	7	20	24	18	19	18	23	17	18
Number of hospitals	75	69	72	84	83	64	48	63	73
Number of arthroplasties	9,670	10,956	18,948	261,540	183,964	91,159	4,435	9,022	12,624

SAP = systemic antibiotic prophylaxis; THA = total hip arthroplasty; TKA = total knee arthroplasty; HA = hemiarthroplasty of the hip.

3,388 (1.1%) reoperations for PJI were reported for the first postoperative year. 93% of the primary arthroplasties had complete 1-year follow-up (7% of the cases had their arthroplasty in 2023, hence did not have full 1-year follow-up. This did not influence our findings). The 1-year incidence of reoperation for PJI was 1.0% for 1 dose of SAP, 0.9% for 2 doses, 0.9% for 3 doses, and 1.1% for 4 doses (Figure 1). The 1-year incidence of reoperation for PJI was 1.2% for cefalotin, 1.1% for cefazolin, 0.8% for cefuroxime, 0.9% for cloxacillin, and 0.9% for clindamycin (Figure 1).

### Primary outcome

**Figure 3:**
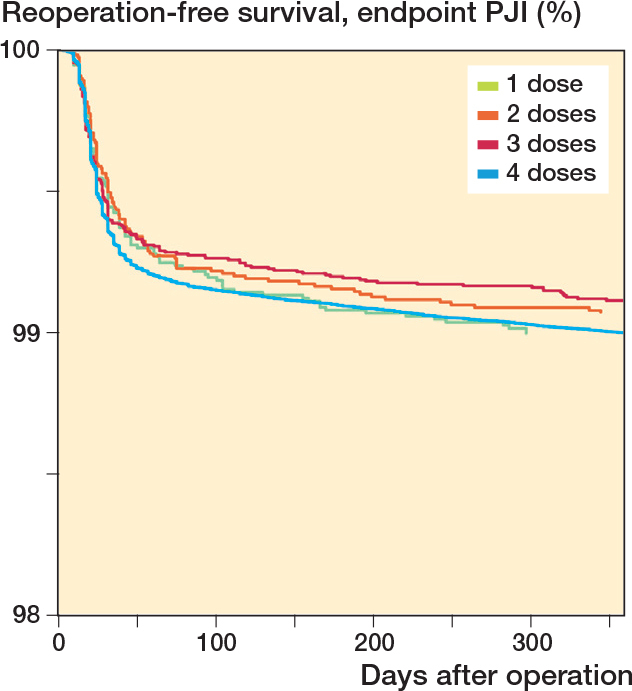
Number of doses of systemic antibiotic prophylaxis, with 1-year reoperation for PJI as endpoint. Cox survival curves adjusted for sex, age, indication for arthroplasty, type of antibiotic, and year of primary arthroplasty.

The 1-year risk and rate of reoperation for PJI was similar for 1, 2, and 3 doses (Table 2 and Figure 3).

**Table 2:** Distribution of number of doses and type of antibiotic used for systemic prophylaxis, in addition to half-life of the antibiotics, number and incidence reoperated for PJI within 1 year, crude and adjusted 1-year risk (HRR), and crude and adjusted 1-year incidence (rate) of reoperation for PJI. The risks and rates are adjusted for sex, age, ASA class, indication for arthroplasty, year of primary surgery, and type of antibiotics or number of doses

	Arthroplasties n	n (%)	1-year reoperation for PJI			
			Crude HRR (CI)	Adjusted HRR (CI)	Crude rate (CI) %	Adjusted rate (CI) %
Number of doses						
4	261,540	3,003 (1.1)	1	1	1.2 (1.1–1.2)	1.0 (1.0–1.1)
1	9,760	98 (1.0)	0.9 (0.7–1.1)	1.0 (0.8–1.2)	1.0 (0.8–1.2)	1.0 (0.8–1.2)
2	10,956	109 (0.9)	0.9 (0.7–1.0)	0.9 (0.8–1.1)	1.0 (0.8–1.2)	0.9 (0.8–1.1)
3	18,948	178 (0.9)	0.8 (0.7–1.0)	0.9 (0.7–1.1)	1.0 (0.8–1.1)	0.9 (0.7–1.1)
Type of prophylactic antibiotics						
Cefazolin	91,159	993 (1.1)	1	1	1.1 (1.1–1.2)	0.8 (0.8–0.9)
Cefalotin	183,964	2,162 (1.2)	1.1 (1.0–1.1)	1.4 (1.2–1.5)	1.2 (1.2–1.3)	1.1 (1.1–1.2)
Cefuroxime	4,435	35 (0.8)	0.7 (0.5–1.0)	1.0 (0.7–1.4)	0.8 (0.5–1.1)	0.8 (0.6–1.1)
Cloxacillin	9,022	85 (0.9)	0.8 (0.7–1.0)	1.2 (1.0–1.6)	1.0 (0.8–1.2)	1.0 (0.8–1.3)
Clindamycin	12,624	113 (0.9)	0.8 (0.7–1.0)	1.1 (0.9–1.3)	0.9 (0.8–1.1)	0.9 (0.7–1.1)
Total	301,204	3,388 (				

Half–life in minutes: cefazolin 90; cefalotin 45; cefuroxime 70; cloxacillin 30; clindamycin 180.

**Figure 4:**
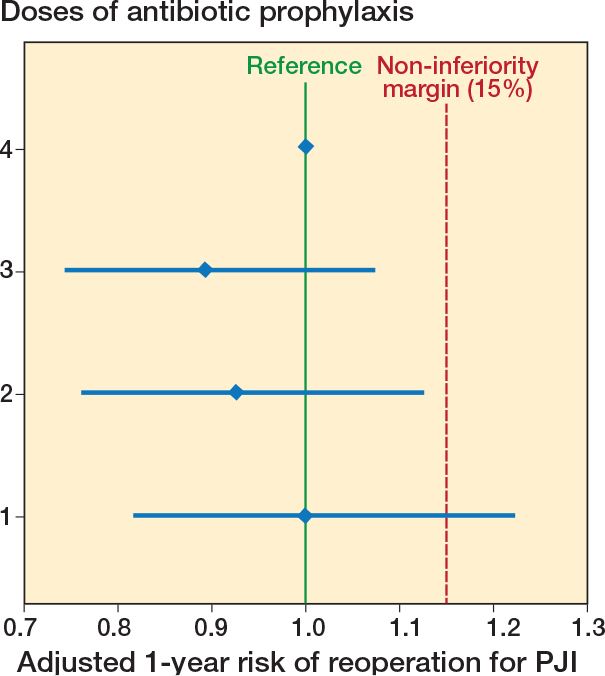
Doses of systemic antibiotic prophylaxis, with 4 doses as reference, with 1-year reoperation for PJI as endpoint with an illustrated 15% non-inferiority limit. Forest plot of Cox risk estimates adjusted for sex, age, indication for arthroplasty, type of antibiotic, and year of primary arthroplasty.

In the non-inferiority analyses, the 1-year risk of reoperation for PJI was non-inferior for 2 and 3 doses of SAP, and neither inferior nor conclusive for 1 dose (Figure 4) compared with the 4-dose regimen. The NNH of the upper margin of the 95% CI for 1 dose was 492, lower than the 667 predetermined by a 15% non-inferiority margin. Hence, the non-inferiority analysis for 1 dose of SAP was inconclusive. For 2 and 3 doses the NNH was higher, with 924 and 1,860 respectively, and thus non-inferior.

Survival analyses after propensity score matching showed similar results for different numbers of doses of SAP, compared with 4 doses (Table 3).

**Table 3:** Propensity score matching of 1, 2, 3, or 1 and 2 doses combined (intervention groups), compared with 4 doses (matched control group ^a^) of systemic antibiotic prophylaxis

Doses compared	Intervention group		Matched control group		1-year risk of reoperation for PJI (CI)	P value
	Included arthroplasties, n	PJIs n	Included arthroplasties, n	PJIs n		
1 vs 4	8,888	89	35,552	342	1.03 (0.82–1.30)	0.8
2 vs 4	15,359	158	61,436	657	0.95 (0.80–1.13)	0.6
3 vs 4	13,568	144	54,272	600	0.95 (0.80–1.14)	0.6
1+2 vs 4	15,359	159	61,436	653	0.97 (0.81–1.15)	0.7

a Matched 1:4 by sex, age, ASA class, indication for arthroplasty, duration of surgery, type of fixation, type of antibiotic, type of arthroplasty, and year of primary surgery, with a tolerance of 0.00001. The Kaplan–Meier (KM) risk estimate had 4 doses as reference and is given with 95% confidence interval (CI) and P value.

**Figure 5:**
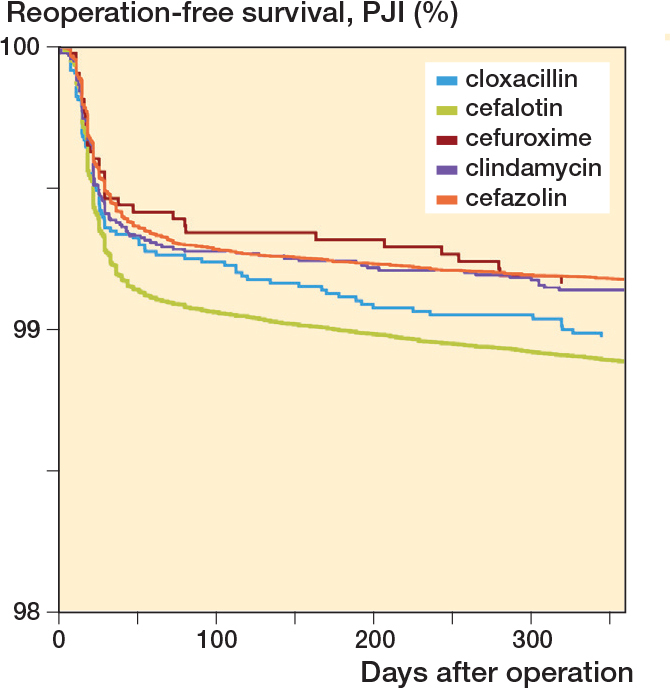
5 most common types of antibiotics used as systemic prophylaxis, with 1-year reoperation for PJI as endpoint. Cox survival curves adjusted for sex, age, indication for arthroplasty, number of doses, and year of primary arthroplasty.

**Figure 6:**
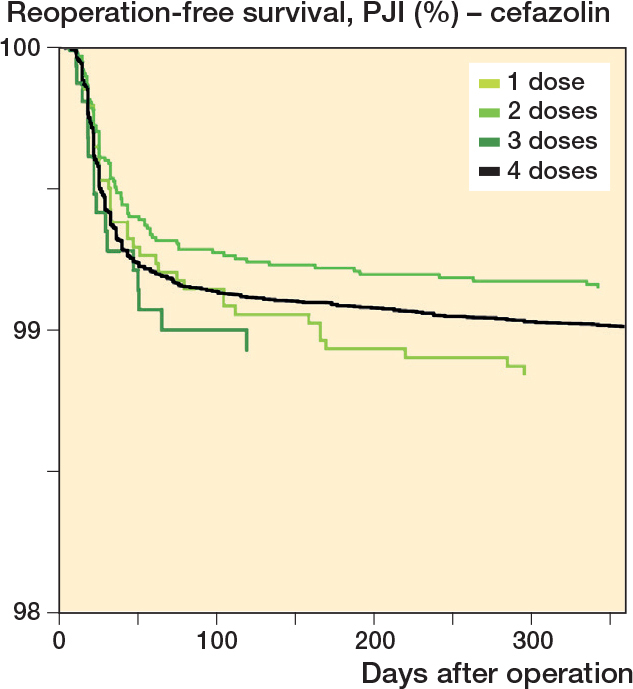
Number of doses of cefazolin, with 1-year reoperation for PJI as endpoint. Cox survival curves adjusted for sex, age, indication for arthroplasty, and year of primary arthroplasty.

Cefalotin (T_1/2_ 45 minutes) and cloxacillin (T_1/2_ 30 minutes) were associated with higher 1-year risk of reoperation for PJI compared with cefazolin (T_1/2_ 90 minutes), whereas the broader spectrum 2nd-generation cephalosporin cefuroxime and, considering the possibility of penicillin or cephalosporin allergy, clindamycin had a similar risk (Table 2 and Figure 5 and 6).

We found a similar risk of reoperation for PJI for 1, 2, 3, and 4 doses of cefazolin (Table 4 and Figure 6).

**Table 4:** Number of arthroplasties with cefazolin as antibiotic prophylaxis, and incidence reoperated for PJI within 1 year, crude and adjusted 1-year risk (HRR), and crude and adjusted 1-year incidence (rate) of reoperation for PJI. The risks and rates are adjusted for sex, age, ASA class, indication for arthroplasty, year of primary surgery

Doses of cefazolin	Included arthroplastie, n	PJIs n	1-year reoperation for PJI		
			Adjusted HRR (CI)	Crude rate (CI) %	Adjusted rate (CI) %
4	77,885	857	1	1.2 (1.1–1.2)	1.0 (0.9–1.1)
1	3,424	39	1.1 (0.8–1.5)	1.1 (0.8–1.5)	1.2 (0.8–1.5)
2	8,842	81	0.8 (0.7–1.1)	1.0 (0.7–1.2)	0.9 (0.7–1.1)
3	1,008	16	1.1 (0.7–1.8)	1.7 (0.9–2.5)	1.1 (0.6–1.6)
Total	91,159	993			

### Secondary outcome

1 dose of SAP was non-inferior (0.90, CI 0.75–1.09) to 4 doses in reoperations for PJI with up to 19 years’ follow-up.

**Figure 7:**
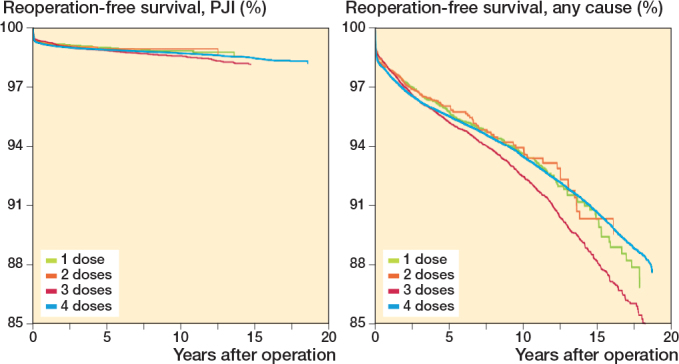
Adjusted Cox survival curves for number of doses of systemic antibiotic prophylaxis, with reoperation for PJI (left panel) or any cause (right panel) as endpoint, adjusted for sex, age, indication for arthroplasty, type of antibiotic, and year of primary arthroplasty.

**Figure 8:**
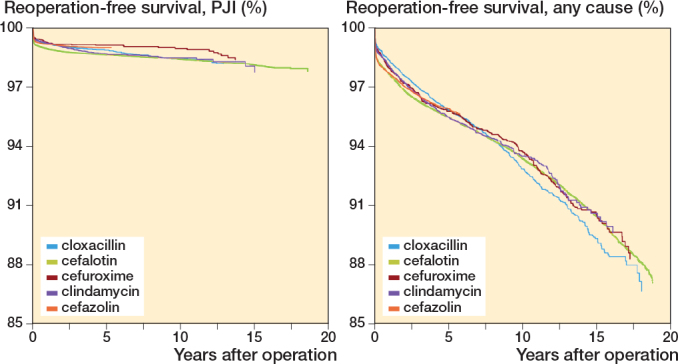
Adjusted Cox survival curves for the 5 most common types of antibiotics used as systemic prophylaxis, with reoperation for (left panel) PJI and (right panel) any cause as endpoint, adjusted for sex, age, indication for arthroplasty, type of antibiotic, and year of primary arthroplasty.

For the secondary endpoint reoperation for any cause with up to 19 years’ follow-up, 3 doses of SAP had slightly higher risk than 4 doses (Table 5 and Figure 7, see Appendix). Otherwise, the findings for reoperation for any cause and PJI with up to 19 years of follow-up were similar to what was found when follow-up was limited to 1 year (Table 5, Figures 8, see Appendix).

**Table 5:** Distribution of number of doses and type of antibiotic used for systemic prophylaxis and adjusted overall (0–19 year) risk (aHRR) of reoperation for any cause or PJI. Adjusted for sex, age, ASA class, indication for arthroplasty, year of primary surgery, and type of antibiotic or number of doses

SAP	Arthroplasties n	Reoperations for any cause		Reoperations for PJI	
		n	Adjusted HRR (CI)	n	Adjusted HRR (CI)
Number of doses					
4	261,540	13,697	1	3,726	1
1	9,760	488	1.0 (0.9–1.1)	115	0.9 (0.7–1.1)
2	10,956	373	0.9 (0.8–1.0)	123	0.9 (0.8–1.1)
3	18,948	1,378	1.2 (1.1–1.2)	290	1.1 (0.9–1.2)
Type of prophylactic antibiotic					
Cefazolin	91,159	2,812	1	1,039	1
Cefalotin	183,964	11,442	1.1 (1.0–1.1)	2,860	1.3 (1.2–1.4)
Cefuroxime	4,435	322	1.0 (0.9–1.2)	51	0.9 (0.7–1.3)
Cloxacillin	9,022	715	1.1 (1.0–1.2)	136	1.2 (1.0–1.5)
Clindamycin	12,624	645	1.0 (0.9–1.1)	168	1.2 (1.0–1.3)
Total	301,204	15,936		4,254	

**Figure 9:**
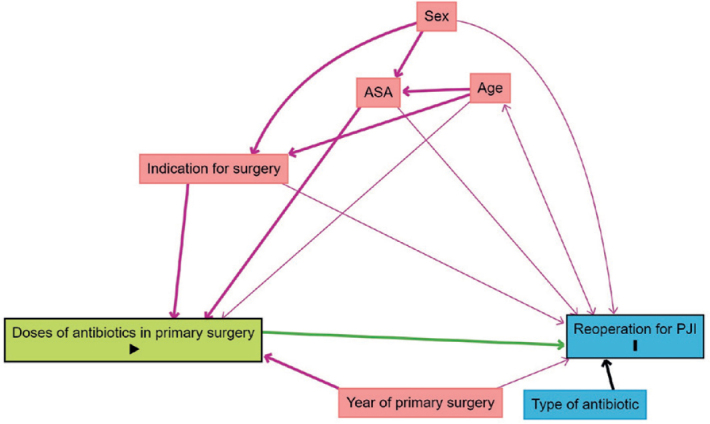
Direct acyclic graph showing relevant confounders for adjustment.

### Sensitivity analysis

In sensitivity analyses, results were similar when restricted to patients with full 1-year follow-up only. In addition, 1-year risk of reoperation for PJI findings was similar for number of doses and type of SAP in frail versus robust patients, long versus normal duration of surgery, THA, TKA, and HA separately, uncemented versus cemented with antibiotic-loaded bone cement, and each type of SAP separately (data not shown). Findings were also similar when adjusted for type of hospital, or with primary hospital as instrument variable (data not shown).

## Discussion

We aimed to investigate, primarily, whether 4 doses of SAP reduced the risk of PJI compared with 1 to 3 doses, and secondarily if there was a difference between types of antibiotics.

We showed that 1, 2, and 3 doses of SAP had a similar risk of reoperation for PJI after primary arthroplasty as 4 doses. In addition, we found a trend towards higher risk of reoperation for PJI when antibiotics with shorter (< 45 minutes) half-lives were used.

We believe that our main finding, that 1 preoperative dose of SAP is sufficient, was robust, even if inconclusive in the non-inferior test due to the results of our sensitivity analyses for frail patients, uncemented arthroplasty and arthroplasty cemented with antibiotic-loaded bone cement, THA, TKA, and HA separately, as well as each type of SAP dosage group separately, and found similar results. We also did not find any beneficial effect of repeated doses in long duration (> 120 minutes) surgery.

### Number of doses of systemic antibiotic prophylaxis

The finding that 1 dose of SAP preoperatively has the same risk for PJI prophylaxis in primary arthroplasty is in accordance with findings from other studies [9,27,28]. A Dutch observational study found no association of type or duration of SAP and the risk of revision for PJI (1- or 2-stage exchange of the prosthesis) [27]. 2 retrospective American single-center studies found similar PJI rates in TKA and THA patients receiving 1 preoperative or multiple doses of SAP [8,9]. A systematic review, including 4 randomized controlled trials, found no benefit of extended postoperative SAP over placebo in preventing surgical site infection [28].

The findings of the present study are in contrast to the findings in the previous NAR study from 2003 [5]. The present study has a considerably larger database, and hence more reoperations for PJI, improved completeness of reporting, and better data quality. We were now also able to adjust for comorbidity and had reoperation for PJI only as endpoint in the main analyses. In 2003, only 46 revisions for PJI were included. We believe that the reporting in general, and of PJI in particular, has improved, making the numbers in the present study more robust and reliable. The focus on PJI has increased, and accordingly the reporting is more correct and complete [19,29].

### Type of antibiotic used as systemic prophylaxis

We found that the 1st-generation cephalosporin, cefazolin, had similar results to the 2nd-generation cefuroxime, in accordance with other trials [30,31].

Cefazolin has been compared with non-cephalosporin antibiotics, showing increased risk for PJI in the non-cephalosporin group [30,31]. However, we found a trend towards a higher risk of reoperation for PJI with the use of cefalotin and cloxacillin, which is accentuated in frail patients. This could indicate a need for repeated doses intraoperatively if antibiotics with short half-lives (< 45 minutes) are used for SAP.

### Antibiotic stewardship and governance

SAP is given to presumably non-infected patients to prevent postoperative PJI or SSI. However, all humans are colonized with abundant commensal bacteria in the microbiota of the skin and gut. Administered antibiotics will apply a selection pressure on this microbiota, and potentially select resistant bacterial strains [14]. A study comparing skin swabs from patients undergoing primary arthroplasty, and subsequent revision, observed an increase in resistance to both cloxacillin (1.9-fold) and gentamicin (4.7-fold) when retested before revision arthroplasty [32]. This is as much a problem for society as for the individual patient, by selecting resistant strains of bacteria, and calls for antibiotic stewardship and governance [10,33]. Using narrow spectrum antibiotics (i.e., 1st-generation cephalosporin), and the lowest dose proven safe (i.e., 1 dose), would be ecologically beneficial in this perspective.

### Cost of postoperatively administered systemic antibiotic prophylaxis

When SAP is administered 4 times on the day of surgery, it must be prepared, administered, and documented, in the operating theatre, in recovery units, and in wards. This is time consuming, laborious, and adds logistical strain and increased cost for the healthcare provider [11]. Therefore, a beneficial effect of having no postoperative dosing of SAP, without compromising outcome, will be saved resources of labor and costs.

### Strengths

The NAR and NHFR have 92–97% completeness of reporting for primary arthroplasties and 88–93% for reoperations, with good granularity and quality of data [19]. The present study is on a national arthroplasty population, with the 3 most common type of arthroplasties (THA, TKA, HA), and includes detailed information on individual patients. PJI after primary arthroplasty is a relatively rare event, and large numbers are required to study differences between groups of patients; even more so with small differences as in a non-inferiority setting. Thus, even though PJI is a rare complication, being large databases, the NAR and NHFR have a relatively large number of reoperations for PJI. Although reported reoperation for PJI is a surrogate endpoint for true PJI, when reported by the surgeon immediately after surgery, it has been found to have quite good sensitivity and accuracy [21,34]. As we included patients from both the NAR and NHFR, we were able to assess whether the findings were valid in different patient groups and for different types of arthroplasties. Thus, the results should have high external validity. In addition, applying different statistical models, resulting in similar results, indicated that the findings were robust. The propensity score-matching analyses resulted in near identical groups and similar results. This indicates good internal validity and minor bias.

### Limitations

We did not have exact information on the timing of preoperative antibiotics. A Swedish study found that only 57% of the patients received adequately timed SAP before TKA [35]. However, timing improved after raising awareness and the introduction of WHO checklists, which has also been widely used in Norway since 2013 [36]. We have no reason to believe that systematic differences in timing of doses of SAP apply bias in our study. Furthermore, we have limited information on time between doses. The reported doses reflect intention to treat and may have been administered otherwise. However, there are strict routines regarding postoperative SAP initiated at end of surgery, thus we have reason to believe that the hospitals have strict routines to follow instructions in the end-of-surgery checklist.

Also, all reported reoperations for PJI will not in fact be due to an infection, as the procedure is reported by the surgeon immediately after surgery before culture results from biopsies are ready. In a validation study from NAR, 9% of reported reoperations for PJI were found to be because of aseptic loosening, prolonged wound discharge, or pain. Likewise, some reoperations reported as aseptic loosening could in fact be a low-grade infection [21].

Unmeasured confounding could interfere with our findings, as inherent confounding applies to register research [37].

### Conclusion

4 doses did not reduce the risk of PJI compared with 1 to 3 doses in primary arthroplasty. Cefazolin, the 1st-generation cephalosporin with the longest half-life, showed the lowest risk of PJI.

Cefuroxime, a 2nd-generation cephalosporin, and the lincosamide clindamycin had similar results to cefazolin, but are less favorable ecologically. Neither repeated doses in general, nor in long-duration surgery, nor in frail patients influenced the risk of reoperation for PJI.

*In perspective*, our findings may improve patient safety and logistics, reduce healthcare costs, and improve antibiotic stewardship if we implement a single preoperative dose of SAP.

## Notes

HD was the initiator of this study and performed the analyses of the data. OL wrote the manuscript. OL and HD contributed to the conception and design of the study. All authors contributed to critical analyses of the data, interpretation of the findings, and critical revision of the manuscript through all stages of the study.

The authors thank all Norwegian surgeons for conscientiously reporting to the NAR and NHFR, and the secretaries, IT analyst, and statisticians at the NAR for entering the data and preparing them for analyses. Thanks are also extended to Prof. Stein Atle Lie and Dr. Tarjei Vinje for statistical inputs. 

Handling co-editors: Keijo Mäkelä and Robin Christensen

*Acta* thanks Harmen B Ettema and Ricardo Sousa for help with peer review of this manuscript.
